# Tag7–Mts1 Complex Activates Chemotaxis of Regulatory T Cells

**DOI:** 10.1134/S1607672922050064

**Published:** 2022-10-27

**Authors:** O. K. Ivanova, T. N. Sharapova, E. A. Romanova, L. P. Sashchenko, D. V. Yashin, G. P. Georgiev

**Affiliations:** Institute of Gene Biology, Russian Academy of Sciences, Moscow, Russia

**Keywords:** regulatory T-lymphocytes, chemotaxis, Tag7, Mts 1

## Abstract

One of the basic features of immune system is the ability to sustain balance between activation and suppression of effector lymphocytes. In this process a key role belongs to the subpopulation of cells called regulatory T cells (Treg). Many cancer and autoimmune diseases are caused by malfunctions of Treg, and investigation of this subpopulation is important for development of new therapeutic approaches. In this study, we demonstrate that regulatory T cells can migrate along the concentration gradient of Tag7–Mts1 complex, and also they produce agents that induce blood cells migration.

In 1996, the gene for the new Tag7 protein (also known as the pepdidoglycan recognition protein 1 PGLYRP1 or PGRP-S) was described in our institute [1]. It belongs to a conserved family of proteins found in various organisms from insects to mammals. These proteins recognize peptidoglycan, a unique component of the bacterial cell wall [2]. In insects, Tag7 was shown to be involved in the antibacterial defense of the body through the activation of Toll receptors [3]. In mammals, this protein is a component of innate immunity, is involved in neutrophil phagocytosis [4], and, in combination with the heat shock protein Hsp70, activates T-lymphocytes and plays an important role in antitumor defense [5].

In addition to Hsp70, Tag7 can bind to the Ca^2+^-binding protein metastasin-1 (Mts1 or S100A4). The most studied function of Mts1 is the involvement in tumor cell metastasis [6]. However, it is also expressed in normal cells of the body, including fibroblasts, lymphocytes, and macrophages. In our laboratory, it was shown that the Tag7–Mts1 complex is able to induce directed migration of human lymphocytes along the concentration gradient (chemotaxis) [7]. This process plays an important role in the development of the immune response, inflammatory reactions, as well as in the pathogenesis of infectious and oncological diseases.

Regulatory T cells (Treg) are a subpopulation of lymphocytes that coordinate the immune response. Their main function is to suppress autoimmune reactions. An imbalance in the maturation and activation of these cells underlies the pathogenesis of diseases such as rheumatoid arthritis, psoriasis, and systemic lupus erythematosus [8]. Regulatory T cells were first described as lymphocytes with the CD4^+^CD25^+^ phenotype that suppress the development of autoimmune diseases in mice [9]. Later, it was found that, for normal maturation and functioning of regulatory T cells, the transcription factor FoxP3 is required. It ensures their suppressive properties by stimulating the expression of *IL2Ra*, *CTLA4*, and *TNFRsf18* genes. FoxP3 also suppresses the expression of effector cytokines (including IL4 and IFNg) and cyclic nucleotide phosphodiesterase 3B (PDE3B) activity, maintaining the necessary homeostasis in regulatory T cells [10]. However, experiments on the identification and sorting of Tregs showed that, among the FoxP3^+^ cells, there are also lymphocytes with the CD4^+^CD25^–^ phenotype. The search for more specific markers for the regulatory T cells led to the discovery of an inverse correlation between the expression of FoxP3 and the IL7 receptor (CD127). It was shown that more than 85% of cells with the CD4^+^CD25^+^CD127^–(Lo)^ phenotype express FoxP3 [11]. In the test of the suppressive properties of CD4^+^CD25^+^CD127^–^ cells, they demonstrated their high efficiency [12]. Thus, the use of these three markers makes it possible to identify regulatory T cells in peripheral blood by flow cytometry with an accuracy of more than 85%. The study of the properties of Treg cells is of great interest in the light of new data on their involvement in the antitumor immune response, wound healing, food allergy, and other body processes.

The aim of this work was to investigate the ability of regulatory T cells for chemotaxis along the concentration gradient of the Tag7–Mts1 complex, as well as to find out whether they secrete this complex into the incubation medium.

Lymphocytes were obtained from the leukocyte mass of healthy donors by centrifugation on a ficoll gradient according to the standard procedure. All donors signed a voluntary consent, the material was taken in the work anonymously. CD4^+^ (Fig. 1) and CD4^+^CD25^+^ (Fig. 2) lymphocyte subpopulations were isolated using kits with Dynabeads magnetic beads (Invitrogen, United States). Chemotaxis experiments were performed in HTS Transwell chambers (96 wells, polyester membrane, 8 µm pores, manufactured by Corning, United States) in serum-free RPMI medium at 37°C, 5% CO_2_, and 95% humidity. The Tag7–Mts1 complex at a concentration of 10^–7^ M and CCL5 (R&D Systems, United States) at a concentration of 5 × 10^–9^ M were used as chemoattractants. To form the complex, Tag7 was mixed with Mts1 in a ratio of 1 : 2 and incubated for 1 h. Serum-free RPMI medium was used as a negative control. Then, 18 h after application to the chemotactic chamber, the number of cells in the lower cell of the chamber was counted using the MTT kit (Abcam, United Kingdom), and the phenotype of migrated cells was determined with a Cytoflex flow cytometer (Beckman Coulter Life Sciences, United States). The following antibodies were used for flow cytometry: antibodies to CD8 and CD4 (Invitrogen, United States), antibodies to CD25 (Biolegend, United States), and antibodies to CD127 (Beckman Coulter, United States). In an experiment with a conditioned medium (Fig. 3), inhibitory antibodies to Tag7 (Invitrogen, United States) and Mts1 (ThermoScientific, United States) was added to the lower cell of the chemotactic chamber 1 h before incubation of the total lymphocytic fraction with supernatants. The proportion of migrated cells for each subpopulation was calculated as (total number of cells in the lower chamber × percentage of this population according to cytofluorometry data)/(total number of cells applied to the upper chamber × percentage of this population in the initial mixture of cells according to cytofluorometry data) × 100% . The figures show the data from at least three independent experiments; data are presented as the mean value ± standard deviation. Data were statistically processed using the SigmaPlot software package (Systat Software Inc, United Kingdom).

**Fig. 1.**
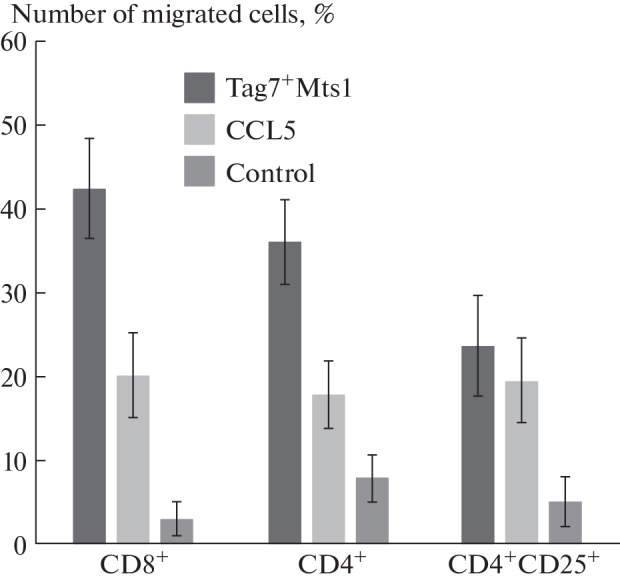
CD4^+^ and CD8^+^ lymphocytes are able to migrate along the concentration gradiant of the Tag7–Mts1 complex and CCL5.

**Fig. 2.**
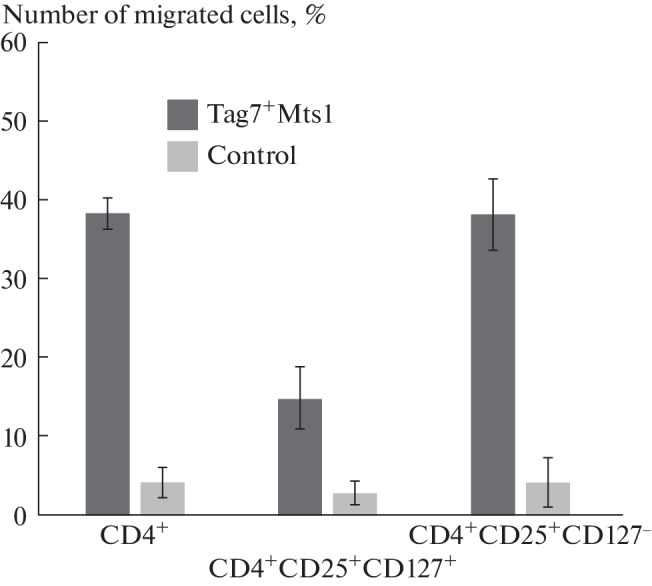
CD127 Treg cells migrate in response to the concentration gradient of the Tag7–Mts1 complex more intensively then the CD127^+^ subpopulation.

**Fig. 3.**
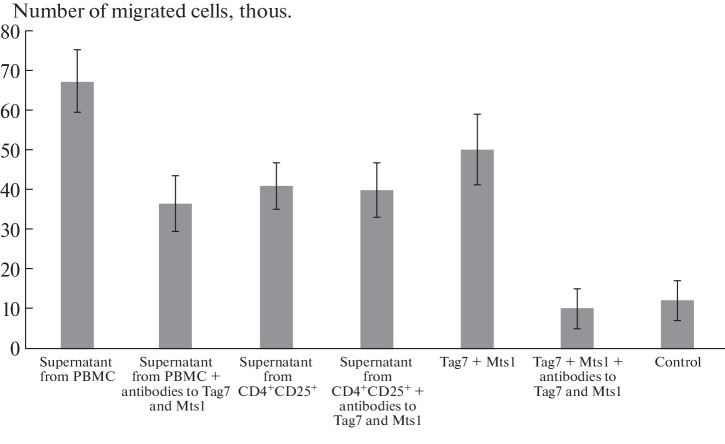
Tag7 and Mts1 do not function as chemoattractants in Treg-induced chemotaxis.

At the first stage of the study, we analyzed the ability of CD4^+^CD25^+^ T cells to migrate along the concentration gradient of chemoattractive substances. To do this, we performed chemotaxis of the total fraction of lymphocytes and determined the phenotype of migrated cells using a flow cytometer. It was previously shown in our laboratory that the Tag7–Mts1 complex induces the migration of lymphocytes [7]. In this work, we used both the Tag7–Mts1 complex and the classical chemokine CCL5 (as a positive control) as chemoattractants (Fig. 3). Both of the used chemotactic agents induced cell migration.

It was found that a significant proportion of CD8^+^ cells actively responded to chemoattractants. Both the CD4^+^ subpopulation and CD4^+^CD25^+^ activated cells also migrated more rapidly in the presence of chemoattractants than without them. Moreover, we observed the migration of CD4^+^CD25^+^ cells both in response to CCL5 and in response to the Tag7–Mts1 complex.

To identify regulatory T cells more accurately, we analyzed the negative expression profile of the CD127 marker. To do this, we preliminarily isolated the fraction of CD4^+^ cells using Dynabeads magnetic beads, applied these cells to a Transwell chamber, and examined the migrated cells with a flow cytometer (Fig. 2). We found a significant difference in the migration of cells expressing and not expressing the CD127 marker. The cells with the CD4^+^CD25^+^CD127^–^ phenotype, which are considered the regulatory T cells (Treg), were significantly more active in response to the concentration gradient of the Tag7–Mts1 complex than the CD127^+^ cells. Thus, we showed that the regulatory T cells are capable of chemotactic migration under the influence of the Tag7–Mts1 complex.

It is well known that one of the most important functions of CD4^+^ cells is the production of various substances that affect other cells of the immune system. To find out whether regulatory T cells produce chemoattractants (in particular, the Tag7–Mts1 complex), we performed an experiment with supernatants. We prepared a medium conditioned with CD4^+^CD25^+^ cells as described in [7] and added it to the lower cell of the chemotactic chamber. The total leukocyte fraction was placed in the upper cell. It is important to note that no pre-activation of the cells was performed. The supernatant from the total fraction of mononuclear cells was used as a positive control (Fig. 3). The medium conditioned with both total leukocyte and CD4^+^CD25^+^ fractions caused migration of a significant number of lymphocytes.

When inhibitory antibodies to Tag7 and Mts1 were added to the supernatants, the activity of lymphocyte migration decreased in the cells where the supernatant from the total lymphocyte fraction served as a stimulant; in the case of the supernatant from CD4^+^CD25^+^ cells, no changes were observed. The results obtained in this experiment indicate that CD4^+^CD25^+^ T cells are able to induce chemotaxis; however, Tag7 and Mts1 are not involved in this process. Previously, it was shown in our laboratory that the Tag7–Mts1 complex is present in the medium conditioned with the total leukocyte fraction [7]. It can be assumed that CD8^+^ cells make the main contribution to the expression of this complex, whereas the regulatory T cells, apparently, do not release it into the incubation medium.

The Tag7–Mts1 complex occupies a special place among chemoattractants. It does not have a tertiary structure that is specific for the classical chemokines (“Greek key”). However, despite this fact, it is able to activate the migration of lymphocytes. We have previously shown that this complex is able to induce migration of NK and T cells. Monocytes and neutrophils showed no ability to migrate in response to the concentration gradient of this complex [7]. In this study, we found that the regulatory T cells with the CD4^+^CD25^+^CD127^–^ phenotype are capable of chemotactic migration along the concentration gradient of the Tag7–Mts1 complex. However, the regulatory T cells themselves apparently do not express this complex but produce other molecules with chemoattractive properties.
